# O “Valor do 0”: Existirá Mais-Valia no Uso do Escore de Cálcio na Estratificação de Indivíduos Sintomáticos com Escore de Zero?

**DOI:** 10.36660/abc.20230105

**Published:** 2023-03-22

**Affiliations:** 1 Centro Hospitalar de Trás-os-Montes Vila Real Portugal S. Cardiologia, Centro Hospitalar de Trás-os-Montes e Alto Douro, Vila Real - Portugal; 2 Universidade da Beira Interior Faculdade de Ciências da Saúde Covilhã Portugal Faculdade de Ciências da Saúde, Universidade da Beira Interior, Covilhã – Portugal

**Keywords:** Cálcio Coronariano, Dor Torácica, Doença das Coronárias, Tomografia Computadorizada por Raios X, Técnicas de Imagem Cardíaca

O escore de cálcio coronariano (ECC) é um bom marcador de risco cardiovascular, com valor prognóstico independente e incremental em relação aos fatores de risco clínicos tradicionais.^1^^-^^4^ É realizado por meio de tomografia computadorizada sem contraste com baixíssima exposição dos pacientes à radiação.

Seu uso agora é recomendado em indivíduos assintomáticos selecionados para refinar a avaliação de risco e ajudar a iniciar ou prolongar farmacoterapias preventivas.^5^

Diferentes estudos relataram taxas anuais de eventos tão baixas quanto 0,06–0,16% em adultos assintomáticos sem ECC detectável.^6^^-^^8^

Embora existam muitas evidências sobre a estratificação de risco em pacientes assintomáticos, o interesse crescente tem aumentado em relação ao comportamento do ECC em pacientes sintomáticos, nos quais a exclusão de doença arterial coronariana obstrutiva pode ser primordial e, por outro lado, pode ter diferentes abordagens e custos, que podem variar entre os países.

O amplo e crescente uso da tomografia computadorizada cardíaca, atingindo também os departamentos de emergência, e seu valor bem estabelecido na estratificação de pacientes com dor torácica também aumentaram o interesse neste campo. Diferentes estudos têm analisado esta questão, relatando baixas taxas de doença arterial coronariana obstrutiva entre pacientes com ECC de zero e baixa incidência de MACE no seguimento neste cenário de pacientes.^9^^-^^11^ Mas também, outros dados da literatura mostram algumas discordâncias ou levantam debates sobre o valor de ECC zero em todos os pacientes, especialmente em sintomáticos mais jovens com presença de fatores de risco pré-teste.^12^

Um estudo recente avaliando o valor do ECC para descartar estenose coronariana em 23.759 pacientes sintomáticos descobriu que o valor diagnóstico adicional de um ECC de zero variava de acordo com a idade. Embora os pacientes com ECC de zero geralmente tivessem uma baixa prevalência de doença coronariana obstrutiva (de 3% a 8% nas diferentes faixas etárias), uma proporção substancial de pacientes com doença arterial coronariana obstrutiva em idade mais jovem também apresentava ECC zero. Os autores referem que uma das explicações é que as lesões ateroscleróticas precoces são geralmente não calcificadas. Em relação a esses resultados, os autores concluíram que o valor diagnóstico adicional de um ECC de zero é altamente dependente da idade, com menor valor para pacientes mais jovens do que para pacientes mais velhos, sendo esse achado observado tanto em homens quanto em mulheres; mas, em todo o espectro de idade, uma proporção substancialmente maior de mulheres com doença arterial coronariana obstrutiva apresentou um ECC de zero em comparação com os homens.^13^

No artigo “Predição de Obstrução Coronariana Significativa em uma População com Suspeita de Doença Coronariana e Ausência de Cálcio Coronariano: Estudos CORE-64/CORE320”^14^ os autores procuraram identificar preditores de obstrução coronariana significativa em pacientes sintomáticos com ECC zero. Um total de 4.258 indivíduos de três coortes foram selecionados para inclusão no estudo. No total, foram incluídos 509 participantes do CORE, dos quais 117 (23%) tinham ECC zero. Desses 509 pacientes, 49% tinham doença arterial coronariana obstrutiva de pelo menos um vaso; 11% dos pacientes com ECC zero também apresentavam doença arterial coronariana obstrutiva de pelo menos um vaso (11 pacientes). Além do tabagismo, outros fatores de risco cardiovascular não se relacionaram significativamente com ECC de zero em pacientes com doença arterial coronariana obstrutiva significativa; ser fumante atual associou-se a um aumento de cerca de 3,5 vezes na chance de obstrução coronariana significativa nesses pacientes. Neste estudo, uma idade mais jovem, sexo feminino e perfil de risco cardiovascular mais baixo foram associados a um ECC zero no cenário de pacientes de alto risco encaminhados para cateterismo cardíaco invasivo. Nesta população, ter um ECC zero foi associado a um risco 83% menor de doença coronariana obstrutiva do que aqueles com qualquer calcificação coronariana. Tabagismo atual foi o preditor mais forte de doença coronariana obstrutiva em pacientes sem cálcio coronariano. Os autores combinaram o tabagismo com a idade e outros fatores de risco para desenvolver um algoritmo que poderia ser proposto para a estratificação do risco de obstrução coronariana significativa em pacientes com ECC zero. No entanto, os resultados mostraram que o valor de discriminação deste algoritmo teve um desempenho limitado, com um poder discriminatório que não alcançou significância estatística na coorte de validação (AUC 0,58, IC 95% 0,43-0,72).

Neste estudo,^14^ os autores tentaram desenvolver um algoritmo para prever doença coronariana obstrutiva significativa em pacientes sintomáticos com ECC zero, mostrando que, apesar de seu potencial interesse, pode ser uma meta desafiadora, possivelmente porque muitos fatores contribuem para sua complexidade e interação entre eles mesmos. O peso da genética, gênero, idade e cada fator de risco cardiovascular (bem como o potencial papel da sua “intensidade” individual) podem contribuir para essa árdua tarefa de refinar o uso do ECC em pacientes sintomáticos. Mais estudos são necessários para avaliar um potencial papel neste cenário.
