# Supplementary material for: O “Valor do 0”: Existirá Mais-Valia no Uso do Escore de Cálcio na Estratificação de Indivíduos Sintomáticos com Escore de Zero?
Source: Arq Bras Cardiol. 2023 Mar 22;120(3):e20230105. [Article in Portuguese] doi: 10.36660/abc.20230105 (PMC10399964; doi:10.36660/abc.20230105)
Supplement: Translation

# Translation: The “Value of 0”: Is There an Added Value in Using the Calcium Score in the Stratification of Symptomatic Individuals with a Zero Score?

**Affiliations:** 1 Vila Real Portugal S. Cardiologia, Centro Hospitalar de Trás-os-Montes e Alto Douro, Vila Real - Portugal; 2 Covilhã Portugal Faculdade de Ciências da Saúde, Universidade da Beira Interior, Covilhã – Portugal

**Keywords:** Coronary Calcium, Chest Pain, Coronary Disease, Computed Tomography, X-Ray, Cardiac Imaging Techniques

Coronary calcium score (CCS) is a good marker of cardiovascular risk, with an independent prognostic value that is incremental over traditional clinical risk factors.^1^^-^^4^It is performed using non-contrast computed tomography with very low radiation exposure to patients.

Its use is now recommended in selected asymptomatic individuals to refine risk assessment and help initiate or prolong preventive pharmacotherapies.^5^

Different studies reported annual event rates as low as 0.06–0.16% in asymptomatic adults without detectable CCS.^6^^-^^8^

While there is much evidence regarding risk stratification in asymptomatic patients, growing interest has been raised regarding CCS behavior in symptomatic patients, in whom excluding obstructive coronary artery disease can be paramount and, on the other hand, may have different approaches and costs, which can vary across countries.

The widespread and growing of cardiac computed tomography reaching the emergency departments and its well-established value in the stratification of patients with chest pain has also raised interest in this field. Different studies have analyzed this issue, reporting low rates of obstructive coronary artery disease among patients with CCS of zero and low MACE incidence at follow-up in this setting of patients.^9^^-^^11^But also, different data from the literature show some disagreements or raise different debates regarding the value of zero CCS in all patients, especially in younger symptomatic with the presence of pre-test risk factors.^12^

A recent study evaluating the value of CCS for ruling out coronary stenosis in 23759 symptomatic patients found that the added diagnostic value of a CCS of zero varied according to age. Although patients with a CCS score of zero generally had a low prevalence of obstructive coronary disease (from 3% to 8% across the different age groups), a substantial proportion of patients with obstructive coronary artery disease at a younger age also had a zero CCS. The authors refer that one of the explanations is that early atherosclerotic lesions are usually non-calcified. Regarding these results, the authors concluded that the added diagnostic value of a CCS of zero is highly dependent on age, with less value for younger than for older patients, being this finding observed in both men and women; but, throughout the age spectrum, a substantially higher proportion of women with obstructive coronary artery disease had a CCS of zero as compared with men.^13^

In the article “Predicting Significant Coronary Obstruction in a Population with Suspected Coronary Disease and Absence of Coronary Calcium: CORE-64 / CORE320 Studies”^14^the authors seek to identify predictors of significant coronary obstruction in symptomatic patients with a CCS of zero. A total of 4,258 individuals from three cohorts were screened for study inclusion. In total, 509 CORE participants were included, of whom 117 (23%) had a zero CCS. Of these 509 patients, 49% had at least one obstructive coronary artery disease; 11% of the patients with a zero CCS also had at least one obstructive coronary artery disease (11 patients). Besides smoking, other cardiovascular risk factors did not relate significantly to CCS of zero in patients with significant obstructive coronary artery disease; being a current smoker was associated with an increase of about 3.5 fold the chance for a significant coronary obstruction in these patients. In this study, a younger age, female gender, and lower cardiovascular risk profile were associated with a zero CCS in the setting of high-risk patients referred for invasive cardiac catheterization. In this population, having a zero CCS was associated with an 83% lower risk of obstructive coronary disease than those with any coronary calcification. Current smoking status was the strongest predictor of obstructive coronary disease in patients without coronary calcium. The authors combined the smoking status with age and the other risk factors to develop an algorithm that could be proposed for risk stratification of significant coronary obstruction in patients with a zero CCS. Nevertheless, the results showed that the discrimination value of this algorithm had a limited performance, with a discriminatory power that did not reach statistical significance in the validation cohort (AUC 0.58, 95% CI 0.43-0.72).

In this study,^14^the authors tried to develop an algorithm to predict significant obstructive coronary disease in symptomatic patients with a zero CCS, showing that, despite its potential interest, it can be a challenging goal, possibly because many factors contribute to its complexity and interaction between themselves. The weight of genetics, gender, age, and each cardiovascular risk factor (as the potential role of its individual “intensity”) can contribute to this hard task of refining the CCS use in symptomatic patients. Further studies are needed to assess a potential role in this setting.
